# Thermotolerance experiments on active and desiccated states of *Ramazzottius varieornatus* emphasize that tardigrades are sensitive to high temperatures

**DOI:** 10.1038/s41598-019-56965-z

**Published:** 2020-01-09

**Authors:** Ricardo Cardoso Neves, Lykke K. B. Hvidepil, Thomas L. Sørensen-Hygum, Robyn M. Stuart, Nadja Møbjerg

**Affiliations:** 1 Department of Biology, August Krogh Building, University of Copenhagen, Copenhagen, Denmark; 2 Data Science Laboratory, Department of Mathematical Sciences, University of Copenhagen, Copenhagen, Denmark

**Keywords:** Ecology, Evolution, Zoology, Environmental sciences

## Abstract

Global warming is already having harmful effects on habitats worldwide and it is therefore important to gain an understanding of how rising temperatures may affect extant animals. Here, we investigate the tolerance to high temperatures of *Ramazzottius varieornatus*, a tardigrade frequently found in transient freshwater habitats. Using logistic modelling on activity we evaluate the effect of 24 hour temperature exposures on active tardigrades, with or without a short acclimation period, compared to exposures of desiccated tardigrades. We estimate that the 50% mortality temperature for non-acclimated active tardigrades is 37.1 °C, with a small but significant increase to 37.6 °C following acclimation. Desiccated specimens tolerate much higher temperatures, with an estimated 50% mortality temperature of 82.7 °C following 1 hour exposures, but with a significant decrease to 63.1 °C following 24 hour exposures. Our results show that metabolically active tardigrades are vulnerable to high temperatures, yet acclimatization could provide a tolerance increase. Desiccated specimens show a much higher resilience—exposure-time is, however, a limiting factor giving tardigrades a restricted window of high temperature tolerance. Tardigrades are renowned for their ability to tolerate extreme conditions, but their endurance towards high temperatures clearly has an upper limit—high temperatures thus seem to be their Achilles heel.

## Introduction

The Tardigrada is a phylum of free-living, microscopic, aquatic invertebrates, which are distributed worldwide in marine, freshwater and terrestrial microhabitats^1–3^. Approximately 1300 described species are accommodated within two major clades–Heterotardigrada and Eutardigrada (including the newly erected Apotardigrada)–while the validity of Mesotardigrada, containing a single thermotolerant species, *Thermozodium esakii* has been questioned^4–9^. Tardigrades are aquatic animals that need to be surrounded in a film water to be in their active feeding and reproducing state. Species living in terrestrial microhabitats with temporary freshwater access (e.g. moss cushions or roof gutters) are referred to as limno-terrestrial^1^. These species endure periods of desiccation by entering the so-called “tun” state. The ability of tardigrades to tolerate severe environmental conditions is well known^10–14^. Indeed, many tardigrades have the ability to enter cryptobiosis*—*a reversible ametabolic state common especially among limno-terrestrial species. Specifically, selected environmental cues may induce one of five subtypes of cryptobiosis: anhydrobiosis (desiccation), anoxybiosis (oxygen depletion), chemobiosis (high toxicant concentrations), cryobiosis (extremely low temperatures) and osmobiosis (high solute concentration)^10,14–17^.

Anhydrobiosis has been intensively studied in tardigrades as well as other organisms^12,18–20^. This physiological state entails striking modifications in anatomy^21–25^. Briefly, during dehydration and entrance into the anhydrobiotic state, tardigrades contract longitudinally, while withdrawing their legs, forming a “tun”^10,22,26^. Our previous investigations suggest that muscle protein filaments play a crucial role in sustaining structural integrity, stabilizing the ametabolic tun, which is essential for resumption of life following rehydration^22^. It has furthermore been suggested that intrinsically disordered “tardigrade unique” proteins are important for structural stabilization during desiccation^27–29^. The latter proteins, however, seem to be restricted to the eutardigrade lineage indicating that anhydrobiosis in general cannot be solely attributed to these proteins^30^. Importantly, tardigrades show an extraordinary tolerance towards extreme levels of radiation, a tolerance that has been directly linked to their anhydrobiotic capabilities^31^. It was recently shown that the tardigrade damage suppressor protein Dsup, which seemingly can protect human cells from radiation damage, binds to nucleosomes protecting chromosomal DNA from reactive hydroxyl radicals^29,32^. All tardigrades seem to have a comprehensive number of genes encoding proteins involved in antioxidant defense^30^.

Here, we focus on the tolerance of tardigrades to high temperatures. Studies on high temperature tolerance in tardigrades started as early as the mid-19^th^ century by Doyère^33^ and Pouchet^34^. While the former found that the limno-terrestrial eutardigrade *Macrobiotus hufelandi* could tolerate temperatures as high as 120–125 °C for a few minutes, the latter author revealed that the tardigrades he investigated could survive temperatures of only ca. 80 °C. A later study by Rahm^35^ corroborated Doyère’s results showing that tardigrades could endure temperatures of 110–151 °C for 30 min. Further studies on this subject were not performed until the early 2000s. In a study on the survival abilities of the limno-terrestrial *Richtersius* (*Adorybiotus*) *coronifer*, Ramløv and Westh^36^ reported that this tardigrade was able to survive temperatures of approx. 70 °C for 1 hour in an anhydrobiotic state. However, exposure to higher temperatures significantly decreased survival and not a single specimen survived exposure to 100 °C.

Subsequent studies on thermotolerance, performed under different methodological approaches (e.g., different exposure time, use of acclimation, etc.), revealed minor differences between tardigrade species^37–40^. Hengherr and colleagues^38^ investigated thermotolerance of the anhydrobiotic state in nine limno-terrestrial tardigrade species (both heterotardigrades and eutardigrades). In accordance with the older studies dating back to the 19^th^ century, exposure to heat-shocks up to 80 °C for a short period, i.e., 1 hour, revealed a moderate decrease in survival. However, temperatures higher than 80 °C resulted in a drastic increase of mortality. Specifically, the most tolerant species in this investigation, the apotardigrade *Milnesium tardigradum*, showed a sharp decrease in survival at temperatures above 100 °C, with almost all specimens dying at temperatures above 103 °C. In contrast to the above mentioned studies on anhydrobiotic tardigrade tuns, recent studies have involved active state individuals of the eutardigrades *Macrobiotus harmsworthi* (limno-terrestrial), *Borealibius zetlandicus* (limnic) and *Halobiotus crispae* (marine) and demonstrated a striking decline in survival at temperatures above 28–30 °C with 100% mortality (LTmax) at 38 °C, 37 °C and 36 °C, respectively^37,39,40^. The temperature required to achieve 50% mortality (LT50) was reported at 33 °C for *B. zetlandicus* and 29 °C for *H. crispae*. In line with these investigations, a study on the pan-Antarctic eutardigrade, *Acutuncus antarcticus*, revealed a LT50 of 36.2 °C and LTmax of 39 °C^41^.

It is presently unknown how desiccated tardigrades handle exposures to high temperatures for periods exceeding 1 hour. Here, we investigate thermotolerance in the temperate limno-terrestrial eutardigrade *Ramazzottius varieornatus*—a strong cryptobiote frequently found in transient freshwater habitats (e.g. roof gutters). As holds for all tardigrades residing in terrestrial habitats, *R. varieornatus* is only active when covered with a film of water. The species, however, readily enters the tun state and in this state tolerates prolonged periods of desiccation. We evaluate the effect of exposures to high temperature in active as well as desiccated tardigrades. We further investigate the effect of a brief acclimation period on active state animals. Based on our data, we estimate logistic models, with tardigrade activity 48 hours after heat shock as the response variable, acclimation and desiccation as factor variables, and temperature as either a factor or a continuous explanatory variable. Based on our models with temperature as a continuous variable we estimate the median lethal temperature, i.e., the temperature required to achieve 50% mortality for active state tardigrades as well as tardigrades in the desiccated tun state.

Our data show that the extremotolerant *R. varieornatus* is vulnerable to high temperatures, although desiccated specimens have a much higher resilience towards high temperatures than active state specimens. These observations are in agreement with previous studies on other tardigrades and we hypothesize that high temperatures destabilize and denature proteins essential to cryptobiotic survival of tardigrades.

## Results

**Figure 1:**
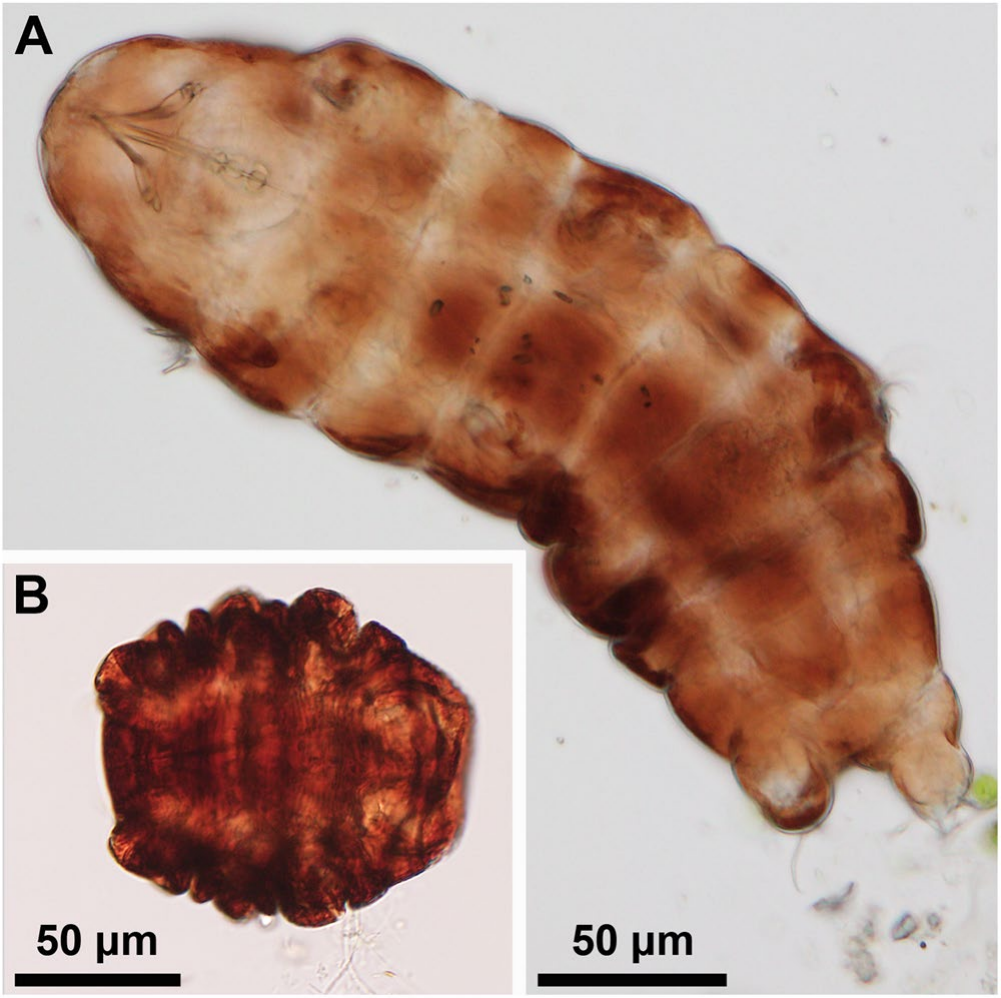
Light micrographs of *Ramazzottius varieornatus* in its active state (**A**) and the cryptobiotic tun state (**B**). During desiccation, an active state tardigrade contracts its body longitudinally and withdraw its legs to form the tun state.

**Figure 2:**
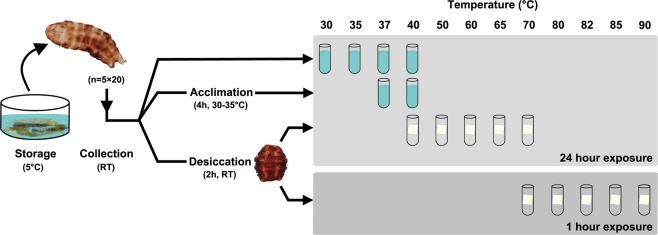
A graphical representation of the methods used to evaluate the effect of exposures to high temperature in active as well as desiccated tardigrades. Active specimens were randomly pooled into groups (5 × ca. 20) at room temperature (RT) and then: (i) exposed to high temperature (30, 35, 37 and 40 °C) for 24 hours or, (ii) briefly acclimated (2 hours at 30 °C followed by 2 hours at 35 °C) and exposed to 37 and 40 °C for a 24 hour period. To assess thermotolerance of anhydrobiotic tuns, tardigrades were also randomly pooled into groups (5 × ca. 20), then desiccated and exposed to high temperature for either 24 hour (40, 50, 60, 65 and 70 °C) or 1 hour (70, 80, 82, 85 and 90 °C).

We estimate survival of desiccated as well as active state *Ramazzottius varieornatus* following exposure to high temperatures (Figs. 1 and 2). Survival estimates are based on the observed proportion of active tardigrades 2 hours, 24 hours and 48 hours after exposure to a given temperature (Fig. 2, Table 1). The proportion of active tardigrades 48 hours after exposure to a given temperature is used as the response variable in the modelling and statistical analyses discussed below.

**Table 1:** Percent active tardigrades (*Ramazzottius varieornatus*) following exposure to various temperatures in either the active or desiccated tun state.

Temp. → Exposure ↓	5 °C ctrl	RT* ctrl	30 °C	35 °C	37 °C	40 °C	50 °C	60 °C	65 °C	70 °C	80 °C	82 °C	85 °C	90 °C
Active, non- acclimated 24 hour exposure	2h: 100.0±0.0 24h: 98.1±1.1 48h: 99.0±1.0		2h: 99.0±1.0 24h: 100.0±0.0 48h: 100.0±0.0	2h: 96.0±1.0 24h: 95.0±1.6 48h: 95.0±0.0	2h: 65.0±5.0 24h: 51.0±3.7 48h: 54.0±4.8	2h: 0.0±0.0 24h: 0.0±0.0 48h: 0.0±0.0								
Active, acclimated 24 hour exposure	2h: 100.0±0.0 24h: 99.0±1.0 48h: 100.0±0.0				2h: 62.5±9.2 24h: 53.6±13.9 48h: 72.2±4.4	2h: 0.0±0.0 24h: 0.0±0.0 48h: 0.0±0.0								
Tun, 24 hour exposure		2h: 97.0±2.0 24h: 97.0±2.0 48h: 97.0±1.2				2h: 97.9±1.3 24h: 99.0±1.0 48h: 98.1±1.2	2h: 96.1±1.8 24h: 94.1±1.8 48h: 91.1±1.9	2h: 93.9±1.9 24h: 92.9±2.5 48h: 92.9±2.5	2h: 8.0±6.8 24h: 27.7±10.0 48h: 37.6±13.5	2h: 0.0±0.0 24h: 0.0±0.0 48h: 0.0±0.0				
Tun, 1 hour exposure		2h: 94.0±1.9 24h: 91.0±1.9 48h: 89.1±1.0								2h: 98.0±1.2 24h: 97.3±3.2 48h: 94.4±3.7	2h: 94.0±3.7 24h: 88.9±3.8 48h: 89.0±2.5	2h: 76.0±6.6 24h: 75.0±6.5 48h: 78.0±3.4	2h: 0.0±0.0 24h: 0.0±0.0 48h: 0.0±0.0	2h: 0.0±0.0 24h: 1.9±1.9 48h: 1.9±1.9

Tardigrades were pooled into 5 replicate groups each containing ca. 20 specimens (approx. 100 tardigrades per experiment or control) and exposed to high temperatures for either 1 or 24 hours. Scoring of activity was performed 2 hours, 24 hours and 48 hours after exposure to the given temperatures and following rehydration (if applicable) and retransfer to 5 °C. Control groups were kept either refrigerated (active state) or at room temperature (desiccated tun state). Data are presented as mean ± se percent active tardigrades. Ctrl= control.

*Desiccated tardigrades kept at room temperature (ca. 23 °C).

### Thermotolerance of active state tardigrades

**Figure 3:**
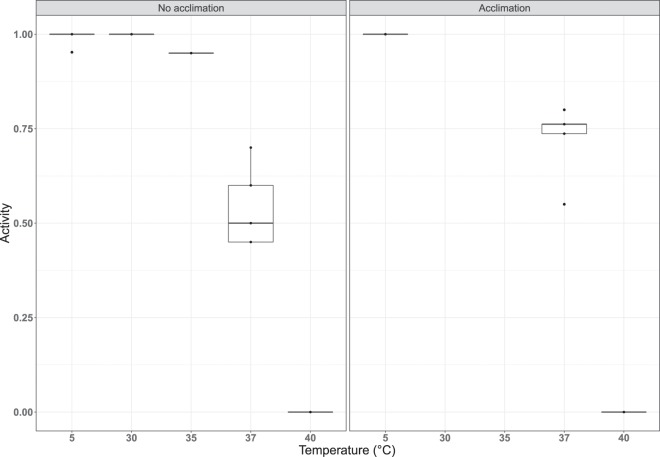
Tardigrade activity (proportion of active animals) following exposure to high temperatures, with and without acclimation. Tardigrades were pooled into 5 groups each containing ca. 20 specimens. Observed data points (•), representing the proportion of active tardigrades in each group, are presented together with medians (horizontal lines), interquartile ranges (boxes), and 1.5*interquartile ranges (whiskers).

**Figure 4:**
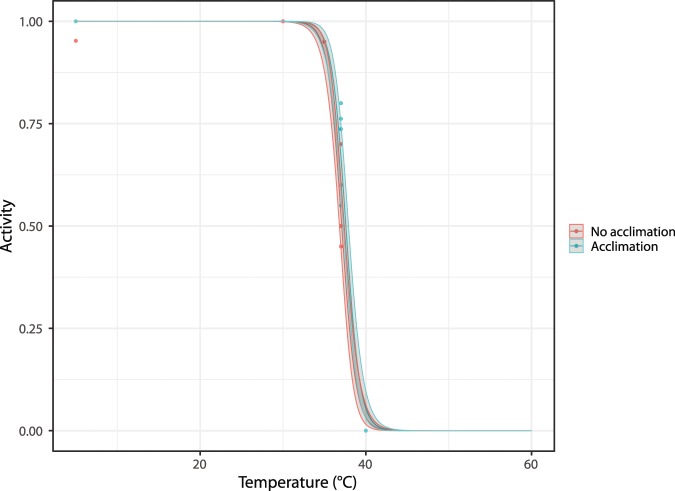
Logistic model of tardigrade activity (proportion of active animals) following exposure to high temperatures, with and without acclimation (see Fig. 2). Observed data points (•), representing the proportion of active tardigrades in each group, are presented together with the model estimate and 95% prediction intervals.

In order to investigate the thermotolerance of the active, feeding *R. varieornatus*, active state tardigrades were exposed to four different temperatures, i.e. 30 °C, 35 °C, 37 °C and 40 °C, respectively, for 24 hours and subsequently retransferred to 5 °C (i.e., the temperature at which the specimen sample was kept before the start of the experiment) – see Fig. 2. As controls, five replicate groups were kept at 5 °C for the entire experiment. The mean ± se activity in proportion of the tardigrades decreases following exposure to 37 °C, and all tardigrades were inactive after being exposed to 40 °C (Table 1). We subsequently inserted a small acclimation step, leaving the tardigrades for 2 hours at 30 °C and then 2 hours at 35 °C, prior to exposure to temperatures of either 37 °C or 40 °C, and found an increase in the survival 48 hours after exposure for the specimens exposed to 37 °C (Table 1). Indeed, the proportion surviving 48 hours after exposure without acclimation was 54.0 ± 4.8%, while with acclimation it was 72.2 ± 4.4%. Figure 3 shows the proportion of specimens active 48 hours after exposure as a function of the exposure temperature and whether or not the tardigrades were acclimated. The proportion of active specimens is significantly affected by the temperature they are exposed to (χ^2^(1) = 568.01, p < 0.01), as well as whether or not they were acclimated before being exposed to high temperatures (χ^2^(1) = 7.89, p < 0.01). Parameter estimates for the logistic regression model with acclimation status and temperature (taken as a continuous variable) are given in Table 2, along with standard errors and significance level. From these, we estimate that the median temperature required to achieve 50% mortality among the specimens that were not acclimated is 37.1 °C, while it is 37.6 °C for the specimens that were acclimated. Model predictions, along with confidence intervals, are shown in Fig. 4.

**Table 2:** Logistic modelling of tardigrade activity following exposure to high temperatures of active state.

${\beta }_{0}$ (Intercept)	43.695 (3.78)***
${\beta }_{1}$ (coefficient of temperature)	−1.178 (0.10)***
${\beta }_{2}$ (coefficient of acclimation)	0.584 (0.26)***

Parameter estimates for the logistic regression model for tardigrade activity with acclimation status and temperature. Standard errors provided in brackets (compare to Fig. 4).

*** Indicates significance at the p < 0.001 level.

### Thermotolerance of desiccated tardigrades

**Figure 5:**
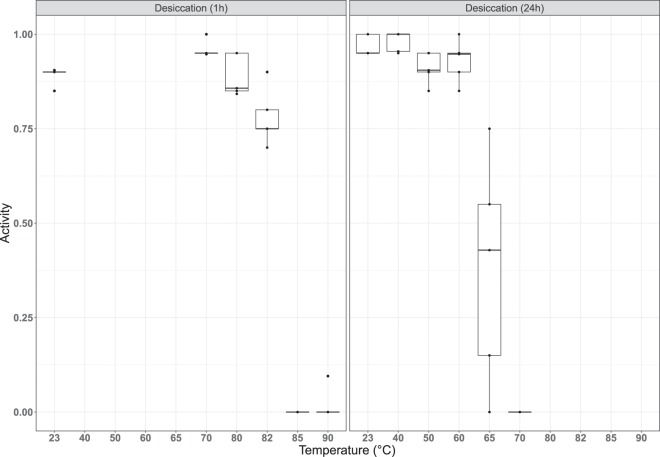
Tardigrade activity (proportion of active animals) following exposure to high temperatures after a period of desiccation (1 hr or 24hrs). Tardigrades were pooled into 5 groups each containing ca. 20 specimens. Observed data points (•), representing the proportion of active tardigrades in each group, are presented together with medians (horizontal lines), interquartile ranges (boxes), and 1.5*interquartile ranges (whiskers).

**Figure 6:**
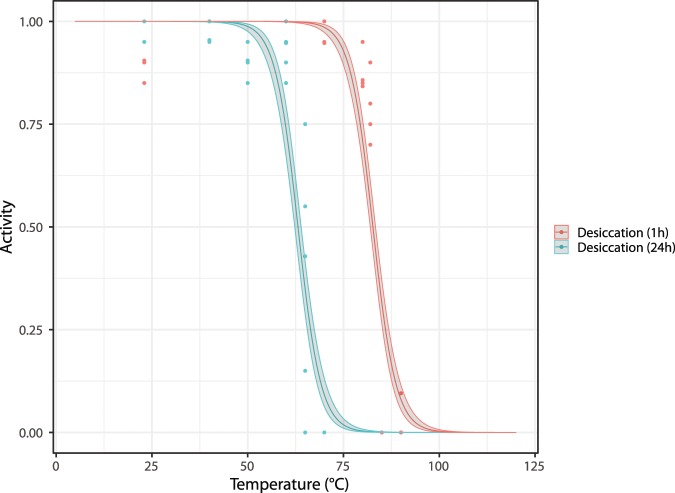
Logistic model of tardigrade activity (proportion of active animals) following exposure to high temperatures after a period of desiccation (1 hour or 24 hours; see Fig. 2). Observed data points (•), representing the proportion of active tardigrades in each group, are presented together with the model estimate and 95% prediction intervals.

Our approach to assess temperature tolerance of anhydrobiotic tuns (i.e., desiccated tardigrades, Fig. 1B) was subdivided into two series: one to test short-duration exposure (1 hour) and one to test longer duration exposure (24 hours). As described in Methods, specimens used in this approach were kept at 5 °C before the start of the experiment, desiccated at room temperature and then exposed to experimental temperatures (Fig. 2). In Fig. 5 we show the proportion of specimens active 48 hours after heat exposure and subsequent rehydration as a function of exposure temperature and exposure time (i.e., 1 hour or 24 hours). Based on this data we found that the survival of desiccated specimens heated to 70 °C is strongly affected by the length of time that they were exposed: 94% of all specimens exposed for 1 hour were active after 48 hours, while 0% of those exposed for 24 hours were active. This difference is clearly highly significant (χ^2^(1) = 177.16, p < 0.01). The logistic regression model using the data on all desiccated specimens finds that both exposure time of the desiccated specimens and temperature (taken as a continuous variable) are highly significant (χ^2^(1) = 100.61, p < 0.01 for exposure time; χ^2^(1) = 416.05, p < 0.01 for temperature). Parameter estimates for this model are given in Table 3 with standard errors and significance level. Based on our data we estimate that the median temperature required to achieve 50% mortality among the specimens that were exposed during 1 hour is 82.7 °C, while it is 63.1 °C for the specimens that were exposed for 24 hours. Model predictions, along with confidence intervals, are shown in Fig. 6.

**Table 3:** Logistic modelling of tardigrade activity following exposure to high temperatures of the desiccated tun state.

${\beta }_{0}$ (Intercept)	27.547 (1.96)***
${\beta }_{1}$ (coefficient of temperature)	−0.333 (0.02)***
${\beta }_{2}$ (coefficient of desiccation)	−6.516 (0.49)***

Parameter estimates for the logistic regression model with desiccation status and temperature, with standard errors provided in brackets (compare to Fig. 6).

*** Indicates significance at the p < 0.001 level.

## Discussion

The outstanding survival capacity of tardigrades has rendered them fascinating for scientists in general and it is well known that the anhydrobiotic tun has an extraordinary tolerance towards a range of environmental stressors^12,16^. This includes, e.g., prolonged exposure to very low sub-zero temperatures as well as exposure to space in low Earth orbit^42–44^. A number of bioprotectants have been proposed to be involved in desiccation tolerance and anhydrobiotic tun formation, protecting membranes and proteins against damage. Bioprotectants may include disaccharides, such as sucrose and trehalose, that replace water during dehydration, thereby stabilizing proteins, nucleic acids and membrane lipids, but presumably also immobilize macromolecules and stabilize cellular structure through vitrification^20,45–47^. In addition, heat shock and LEA (Late Embryogenesis Abundant) proteins may play a protective role (e.g., acting as molecular shields and chaperones) and/or be involved in the repair process after desiccation^36,48–52^. More recently, other proteins such as the tardigrade-unique intrinsically disordered proteins have been associated with anhydrobiotic survival^27,53–57^. We propose that muscle protein filaments play a crucial role in sustaining structural integrity of the anhydrobiotic tun state and further hypothesize that a highly effective antioxidant defense apparatus as well as high fidelity transcription-coupled DNA repair are crucial for post-cryptobiotic survival^22,30,51^.

In the current study we present analyses on high temperature tolerance in active specimens and anhydrobiotic tuns of the limno-terrestrial tardigrade *Ramazzottius varieornatus*. Our data show that anhydrobiotic tardigrade tuns tolerate much higher temperatures than the active state, which corroborates previous observations^38^, but at the same time our results emphasize that exposure time matters. Indeed, the median temperature required to achieve 50% mortality dropped from 82.7 °C following 1 hour exposures to 63.1 °C after 24 hour exposures.

Our results on 1 hour exposures of *Ramazzottius varieornatus* tuns to high temperatures are comparable with those reported in previous studies on other tardigrades^36,38^. In *R. varieornatus* the proportion of active tardigrades 2 hours after heat shock at 80 °C and following rehydration is ca. 94%. Similar survival proportions were found for a number of tardigrade species investigated by Hengherr *et al*., such as *Macrobiotus sapiens* (80.8%) and *Milnesium tardigradum* (97.5%)^38^. Importantly, 91.7% of the latter apotardigrade species were reported to survive exposure to 100 °C, whereas *R. varieornatus*, under the current experimental conditions, could not survive exposure to 85 °C. Other tardigrade species appear less tolerant than *R. varieornatus*. For instance, 56.7% of the heterotardigrade *Echiniscus testudo* survived exposure to 80 °C, whereas only 17.5% of eutardigrades *Macrobiotus tonollii* and 20% of *Richertsius* (*Adorybiotus*) *coronifer* survived similar exposures^36,38^. Given this discrepancy observed among tardigrades, a characterization of genomes and expression profiles of the species studied could provide important new perspectives on how tolerance to high temperatures is achieved and whether distinct differences exist among evolutionary lineages.

Investigations on temperature tolerance of tardigrades in the active state, i.e. specimens that have not been allowed to form the anhydrobiotic tun, reveal much lower tolerance levels as compared to the tuns, with a reported 100% mortality between 36 °C and 39 °C, depending on the species^37,39–41^. Interestingly, the estimated 50% mortality temperature of 37.1 °C for the active state of *R. varieornatus* is not far from the currently measured maximum temperature in Denmark, i.e. 36.4 °C^58^. Our data, however, provide evidence that active state tardigrades have the potential to acclimate to higher temperatures. Specifically, we observed a small, but significant increase in 50% mortality temperature from 37.1 °C to 37.6 °C in non-acclimated and briefly acclimated tardigrades, respectively. Accordingly, we hypothesize that acclimatization in natural habitats may provide active state tardigrades the ability to tolerate rising temperatures. Nevertheless, tardigrades — and especially active state specimens — are clearly sensitive to high temperatures, which seem to be an Achilles heel for their otherwise extraordinary tolerance towards extreme environmental conditions. We hypothesize that high temperatures destabilize and denature proteins essential for their cryptobiotic survival. The vulnerability of tardigrades towards high temperatures clearly underlines the difference between extremophile prokaryotes and extremotolerant metazoans, such as tardigrades — a distinction that has been recognized by other authors^29,59^. Extremophile bacteria and archaea have adapted to life in high temperature environments with optimal growth temperatures depending on thermostable proteins (such as the *Taq*-polymerase; reviewed in, e.g.^60^). Tardigrades survive extreme conditions by entering cryptobiosis — a state of extreme dormancy, where metabolism comes to a reversible stop. Cryptobiotic survival in turn depends on the organism sustaining structural integrity and our investigations indicate that actin filaments are crucial for stabilizing the three-dimensional structure of the anhydrobiotic state^22,25,61^. In effect, we propose that ATP dependent “*rigor mortis*” ensure tardigrade structure during anhydrobiosis.

Resistance to desiccation and brief exposure to high temperatures are found in a number of other invertebrates. Various groups of small, planktonic crustaceans such as Copepoda possess at least one dormant form during their ontogenesis, which could be the egg, a larval stage or even the adults (reviewed in^62^). It should be noted that dormancy as found among copepods is more similar to diapause and encystement in tardigrades than to cryptobiosis, while other organisms (such as bdelloid Rotifera) share tardigrades’ ability to enter anhydrobiosis at any ontogenetic stage^13,63,64^.

The cysts of the anostracan crustaceans *Artemia franciscana* and *Branchiopus schaefferi* as well as adult rotifer *Philodina roseola* are able to endure temperatures up to 130 °C during a 10 minutes-long exposure, at least during a slow heating exposure^65^. The resistance that characterizes *Artemia* cysts seems to be dependent on high trehalose concentrations, the heat shock protein and molecular chaperone p26, and an RNA-binding protein (Artemin) with RNA chaperone activity (reviewed in^66^). Likewise, the occurrence of a small heat shock protein with chaperone activity, the antoxidant ferritin and LEA proteins were identified in resting eggs of the monogonont rotifer *Brachionus plicatilis*^67^. In the related species *Brachionus manjavacas*, a vitellogenin-like protein and four specific families of heat shock proteins (hsp40, hsp60 and hsp70) also seem to be important for thermotolerance^68,69^. Rather different is the condition found in bdelloid rotifers, as trehalose seems to be absent in these organisms, which lack trehalose biosynthesis genes^70,71^. In this group, dehydration-inducible proteins such as LEAs thus seem to be more relevant for anhydrobiosis^71^.

Nematodes also have the ability to withstand desiccation and survive extreme environmental conditions, including brief exposures to high temperatures^72^. For instance, anhydrobiotic juveniles of the seed gall nematode *Anguina agrostis* survived a 5 minute exposure to 155 °C^73^. This temperature is to our knowledge the highest recorded for any metazoan. In contrast to tardigrades and bdelloid rotifers, which enter a tun, nematodes tend to coil into tight spirals during the dehydration-induced anhydrobiosis^72^. This process seems to be accompanied by accumulation of trehalose, which reaches maximal concentrations at an early stage of dehydration^74^. Preservation of biological integrity in severe desiccation seems to rely on LEA proteins^75^.

As observed for tardigrades, all of the above mentioned invertebrate anhydrobiotes (rotifers, nematodes, etc.) possess a number of bioprotectants that appear to have a role in desiccation tolerance. In addition, we hypothesize that muscle protein filaments likely play a role in maintaining structural integrity of rotifer tuns and nematode spirals as we think they do in tardigrades. It is otherwise challenging to compare the observations obtained from various metazoans with the results we report here. This is mainly because the experimental approach is usually very different, e.g., exposure time in experiments performed with crustaceans, rotifers and nematodes is very short — typically 5–10 minutes — compared to our 1 hour and 24 hour exposure times. However, further investigations on tolerance to heat exposure in all these organisms could provide new insights that would help understand their limits of tolerance more comprehensively. In any case, the brief exposures that are tolerated among the mentioned metazoans underline the difference between extremophile prokaryotes and extremotolerant metazoans. To recapitulate, while extremophile bacteria and archaea have adapted to life in high temperature environments, the metazoans typically tolerate briefer exposures while in a dormant state.

## Methods

### Collection of specimens

The sediment sample containing the specimens of adult *Ramazzottius varieornatus* Bertolani and Kinchin, 1993 (Eutardigrada, Hypsibiidae) used in the current study was collected in February 2018 from a roof gutter in Nivå, Denmark (N 55°56.685′, E 12°29.775′), which freezes during winter and frequently dries out during summers. The sample was frozen under wet conditions and stored at −20 °C for 7 months prior to the experiments. In September 2018, the sample was thawed, diluted in ultrapure water (Millipore Milli-Q^®^ Reference, Merck, Darmstadt, Germany) and examined for specimens of *R. varieornatus* with the help of a stereomicroscope. In order to obtain the amount of specimens necessary for this study (2017 individuals in total), the sample was kept refrigerated at ca. 5 °C and examined recurrently between September and November 2018. Although tardigrades were not cultured *sensu stricto*, the microcosm in which they were kept together with the sediment from the locality (also including moss leaves, plant litter, etc.) provided them with sufficient food. We have maintained tardigrade populations in this way for years in the lab. Before the beginning of the experiments all specimens of *R. varieornatus* were, thus, well-fed and acclimated to 5 °C.

### Experimental procedure

Highly active, adult (parthenogenetic) tardigrades were collected from the sample under a stereomicroscope and transferred to embryo dishes using a Pasteur pipette, at room temperature (21–25 °C). The collected specimens were randomly pooled into groups of ca. 20 tardigrades and used for temperature experiments under the conditions described below (Fig. 2).

#### Assessing thermotolerance of active state tardigrades

Firstly, the temperature tolerance of active specimens was assessed. Specifically, five groups of ca. 20 active tardigrades were transferred to Eppendorf tubes with 1.5 ml of moderately hard reconstituted water^76^ and then exposed to temperatures of 30, 35, 37 and 40 °C using a AccuBlock Digital Dry Bath heating system (Labnet International, Edison, NJ) for approx. 24 hours. The time necessary to increase from room temperature to the experimental temperature ranged between 3–7 minutes. After experimental temperature exposure, the tubes with tardigrades were kept for 1 hour at room temperature and subsequently stored at 5 °C. In addition, five groups of ca. 20 tardigrades were kept refrigerated (5 °C) for 24 hours as controls. A moss leaf was deliberately added in each Eppendorf tube, in order for the tardigrades to have something to hold on to and not spend too much energy searching for substrate^77,78^. We further tested the tolerance of the active state following a brief high temperature acclimation period. In this approach, the groups of ca. 20 specimens spent 2 hours at 30 °C and then 2 hours at 35 °C prior to their 24 hour exposure to temperatures of either 37 or 40 °C. In addition, five replicate groups of ca. 20 tardigrades were kept refrigerated (5 °C) for 28 hours as controls.

#### Assessing thermotolerance of desiccated tardigrades

We then continued to assess the temperature tolerance of the anhydrobiotic tun state. Specifically, five groups of ca. 20 specimens were transferred in a small volume of ultrapure water onto a piece of filter paper (1 × 1 cm) placed inside an embryo dish. The desiccation process was monitored under a stereomicroscope, during 1 hour at room temperature, in order to assure that the tardigrades underwent a proper anhydrobiosis process and formed a tun, while the water evaporated from the filter paper. Afterwards, the embryo dishes with the desiccated tuns on filter paper were transferred to a Sicco mini-vitrum desiccator with silica (relative humidity ranged between 22–36%) for 1 hour. After these initial 2 hours of desiccation, the filter papers holding the tuns were transferred into 0.2 ml PCR tubes, incubated in a C1000 Touch thermal cycler (Bio-Rad, Hercules, CA) and exposed to temperatures of 40, 50, 60, 65 or 70 °C for 24 hours. After experimental temperature exposure, the PCR tubes with tardigrades were kept for 1 hour, at room temperature, before the tardigrades were rehydrated with 2 ml of ultrapure water and kept for 1 hour at RT and then transferred to 5 °C. As controls, five groups of ca. 20 tardigrades were desiccated and transferred into 0.2 ml PCR tubes, as explained above, and then kept inside the desiccator chamber (ca. 23 °C) for 24 hours, before being rehydrated with 2 ml of ultrapure water, kept for 1 hour at RT and transferred to 5 °C. An alternative approach was also used, in which the anhydrobiotic specimens were exposed for only 1 hour to high temperatures: 70, 80, 82, 85 or 90 °C. For this alternative approach, the five groups of ca. 20 tardigrades were otherwise treated as explained above for the 24 hours exposure experiment, and controls were kept inside the desiccator chamber for only 1 hour at ca. 23 °C.

### Assessment of tardigrade activity

The specimens were monitored for a period of 2 days following temperature exposures with activity checks at 2, 24 and 48 hours. The activity level of single specimens was assessed by observing the tardigrades under a stereomicroscope. The tardigrades were considered active, and alive, if clear spontaneous movements of legs, or the main body, were observed or when they were responsive to a gentle tactile stimulus. In contrast, tardigrades were considered inactive if they were not showing spontaneous movements of the body parts, nor responding to the tactile stimulus. As inactive tardigrades are not necessarily dead, the activity data reported may represent a slight underestimation of survival rates.

### Data analyses and statistics

In all assessments tardigrade activity was calculated as the proportion of active tardigrades in each replicate group, i.e. the number of active specimens divided by the total number of specimens in each group. These proportions were used as the data points. As our data are not normally distributed we use non-parametric summary measures (medians and interquartile ranges) to estimate and plot the distribution of tardigrade activity (Figs. 3 and 5). Furthermore, we use logistic regression modelling to test the effects of acclimation and desiccation time on the survival of specimens as described below (Figs. 4 and 6). All analyses were carried out using R: A language and environment for statistical computing^79^, with the ggplot2 package. Light microscopic images of tardigrades were acquired with an Olympus DP22 digital microscope camera mounted on an Olympus BX53 microscope. Final assemblage of graphs and light microscopic images was conducted in Photoshop CS5 version 15.0.0 or CorelDRAW Graphics Suite 2017.

#### The effect of acclimation on the ability to tolerate high temperatures

To test the effect of acclimation on the survival of specimens subsequently exposed to high temperatures, we focus on specimens that were exposed to 3 different temperatures (5, 37, and 40 °C) either with or without being acclimated first. Firstly, we create a logistic regression model with activity after 48 hours as the binary response variable and two factors as fixed effects: acclimation (yes/no), and temperature (5, 37, 40 °C). Secondly, we create another logistic regression model with activity after 48 hours as the binary response variable, again with acclimation as a factor variable, but this time with temperature treated as a continuous variable (i.e., a type of dose-response model). This model expresses the proportion of animals active after 48 hours of rehydration as follows:

$$PropActive{48}_{i}=\frac{1}{1+\exp (-\,({\beta }_{0}+{\beta }_{1}tem{p}_{i}+{\beta }_{2}{A}_{i}))}+{e}_{i}$$

where $PropActive{48}_{i}$ is the proportion of animals in replicate *i* which were observed to be active after 48 hours of rehydration; ${A}_{i}$ is a dummy variable equal to 1 if the specimens in replicate *i* were acclimated and 0 otherwise; and $tem{p}_{i}$ is the temperature that specimens in replicate *i* were exposed to. The parameters ${\beta }_{0}$, ${\beta }_{1}$ and ${\beta }_{2}$ are estimated in order to minimize the sum of squared differences between the model predictions and the observations. These parameters can also be used to give an estimate of the median lethal temperature, i.e. the temperature required to achieve 50% mortality: this is given by $-{\beta }_{0}/{\beta }_{1}$for the specimens that were not acclimated and $-({\beta }_{0}+{\beta }_{2})/{\beta }_{1}$ for the specimens that were acclimated. We validate both models and test the significance of all effects using approximate likelihood ratio tests.

#### The effect of desiccation on the ability to tolerate high temperatures

To test the effect of desiccation time on the survival of specimens subsequently exposed to extreme temperatures, we firstly compare the specimens that were desiccated for 1 hour and then heated to 70 °C with the specimens that were desiccated for 24 hours and then heated to 70 °C. Each of these two groups consists of 5 replicates each with 20 tardigrades. We compare the total proportion active after 48 hours using a Chi-squared test for comparing independent proportions. Secondly, we specify a logistic regression model, this time using the data on all desiccated specimens. Our model has activity after 48 hours as the response variable, desiccation time as a fixed effect (factor) and temperature as a second fixed effect (continuous). This model expresses the proportion of animals active after 48 hours of rehydration as follows:

$$PropActive{48}_{i}=\frac{1}{1+\exp (-\,({\beta }_{0}+{\beta }_{1}tem{p}_{i}+{\beta }_{2}{D}_{i}))}+{e}_{i}$$

where $PropActive{48}_{i}$ is the proportion of animals in replicate *i* which were observed to be active after 48 hours of rehydration; ${D}_{i}$ is a dummy variable equal to 1 if the specimens in replicate *i* were desiccated for 24 hours and 0 otherwise; and $tem{p}_{i}$ is the temperature that specimens in replicate *i* were exposed to. The parameters ${\beta }_{0}$, ${\beta }_{1}$ and ${\beta }_{2}$ are estimated in order to minimize the sum of squared differences between the model predictions and the observations. These parameters can also be used to give an estimate of the median lethal temperature, i.e. the temperature required to achieve 50% mortality: this is given by $-{\beta }_{0}/{\beta }_{1}$for the specimens desiccated for 1 hour and $-({\beta }_{0}+{\beta }_{2})/{\beta }_{1}$ for the specimens desiccated for 24 hours. We again validate the model and test the significance of the desiccation time using approximate likelihood ratio tests.

## Data Availability

Datasets on tardigrade activity generated and analyzed during the current study are available from GitHub (https://github.com/robynstuart/tardigrades).

## Code availability

Computer codes have been uploaded to GitHub (https://github.com/robynstuart/tardigrades).
